# Conceptualization and Assessment of Shame Experience and Regulation: An Umbrella Review of Synthesis Studies

**DOI:** 10.1002/cpp.70136

**Published:** 2025-08-20

**Authors:** Carlo Garofalo, Laura Giammarioli, Irene Aiolfi, Elisa Delvecchio, Claudia Mazzeschi

**Affiliations:** ^1^ Department of Philosophy, Social Sciences and Education University of Perugia Perugia Italy; ^2^ Department of Humanistic Research and Innovation University of Bari Aldo Moro Bari Italy

**Keywords:** emotion, emotion regulation, measurement, psychopathology, shame, shame coping

## Abstract

Shame experience and regulation are of crucial importance in the study of emotion and psychopathology. Considering the variety of conceptualizations and operationalization methods in the shame literature, the present review aimed to provide a meta‐synthesis of current knowledge. First, we examined how shame and shame regulation are conceptualized. Second, we aimed to identify the most used measures of shame experience and regulation. Third, we gauged the literature for levels of consistency between conceptualization and methods of operationalization. An umbrella review of recent (2018–2023) systematic reviews and meta‐analyses was conducted: 17 studies were included, integrating data from a total of 748 samples and approximately 166,172 participants. Data on title, authors, journal, aims of the review, conceptualization and measures of shame and shame regulation, and main findings were extracted. A relative majority of studies (roughly 63%) conceptualized shame as a multidimensional construct, and the most used measure was the TOSCA (Test of Self‐Conscious Affect) in 76% of cases. Only partial coherence between conceptualization and measurements of the constructs of interest was detected in the retrieved literature. Most measures capture different dimensions of shame, but these were not always leveraged in favour of ‘total score’ considerations. Notably, several reviews referred to shame regulation, but only two included measures of shame regulation. These findings stress the importance of increased consistency between conceptual and methodological levels when studying shame and, in particular, shame regulation, in order to aid integration across studies and increase the theoretical solidity and applied usefulness of empirical findings.

## Summary

- Shame and shame regulation are consequential for several forms of psychopathology, although shame regulation and externalizing forms of psychopathology have received less attention in recent synthesis studies.
- Although most conceptual models posit that shame is multifaceted and involves different dimensions, 63% of the included reviews were based on multidimensional conceptualizations, while 37% were based on unidimensional conceptualizations of shame.
- Crucially, even among reviews based on multidimensional conceptualizations, integration of empirical findings was often based on total scores or on selected dimensions of broader construct.
- Increased consistency between conceptual definitions and assessment methods appears needed to aid the clinical translation of empirical research syntheses.

## 1 Introduction

Shame has been defined as the quintessential human emotion (Lewis 1995) and as ‘the master emotion of everyday life’ (Scheff 2000). Along these lines, Tangney et al. (2007) argued that shame is the most painful emotion of everyday life, leading to a sense of uselessness and impotence, as it hinges on evaluation of the deepest self rather than of mere behaviour. However, this description likely reflects the most intense and maladaptive declensions of shame experience; while consideration of milder—conscious or unconscious—forms of shame as well as forms of shame that are adaptive or functional call for more nuanced perspectives (Scheff 2003; Elison et al. 2014). At any rate, shame experience and regulation serve an important role in many aspects of psychological functioning (Schimmenti 2012; Schimmenti and Caretti 2016) and have been associated with several clinical problems (Schoenleber and Berenbaum 2010; Goss and Allan 2009). Different forms of psychopathology have repeatedly been linked to prolonged shame. Interpersonal traumatization and stress are more likely to be experienced when we embrace views of ourselves that are unacceptable; this can lead to depressive paralysis or narcissistic rage (Trumbull 2003). Gaudet et al. (2015) found shame to be negatively correlated with psychological well‐being and considered it a risk factor that could lead to interpersonal violence, depression and post‐traumatic stress disorder. Other studies focused on the role of shame in obsessive–compulsive–related disorder and anxiety (Szentágotai‐Tătar et al. 2020) as well as personality disorders characterized by high levels of trait anxiety (Schoenleber and Berenbaum 2010). Further studies reported that shame may play an important role in the aetiology of, maintenance of and recovery from eating disorders (Goss and Allan 2009; Manjrekar et al. 2013). Several scholars have posited that shame may serve as a significant factor in the psychodynamic underpinnings of suicidal behaviour (Breed 1972; Gaudet et al. 2015). Notably, while depression is often considered a primary precursor to suicide, Breed (1972) suggested that shame can be a direct catalyst or an indirect consequence of inward‐directed anger. Seminal studies have also linked shame and narcissistic personality disorder. Specifically, Zaslav (1998) argued that in grandiose narcissism, individuals defensively deny shame, whereas in vulnerable narcissism, individuals exhibit excessive preoccupation with anticipated negative evaluation, demonstrating heightened sensitivity to perceived criticism or slight. Moral emotions like shame also play a key function in the development and manifestation of aggressive or otherwise antisocial behaviour (Elison et al. 2014; Maggi et al. 2022; Stuewig and Tangney 2007; Velotti et al. 2014).

Considering the host of negative correlates of shame, it is no surprise that research on shame has been a cornerstone of emotion research. As research on shame and its regulation accumulates, gauging their conceptualizations and methods of operationalization—as well as consistency between the two—represents an important endeavour to take stock of existing theories and assessment measures and move the field forward. In the present study, we first review relevant theory on the nature of shame and its regulation, highlighting their relevance for adaptive and maladaptive psychological functioning. Then, we present the results of an umbrella review of synthesis studies, aimed to integrate ways in which shame and shame regulation are conceptualized and measured.

### 1.1 Shame as Functional and Dysfunctional Emotion

Although shame has been linked to a variety of negative correlates in the psychopathology domains, this is not to say that shame is, for all intents and purposes, *negative*. In fact, emotions have evolved to fulfil survival, reproductive and even social goals. However, while basic emotions can serve the above‐mentioned purposes, self‐conscious emotions seem to be particularly concerned with social purposes: In particular, shame has evolved for acquiescent purposes and is able to elicit commiseration in spectators (Tracy and Robins 2004). Although shame is often considered a negative emotion, it can serve both negative and positive functions. Indeed, while many studies support the link between shame and maladaptive correlates, theory and research provide a more nuanced picture, suggesting that shame can be a functional emotion; thus, it would be more accurate to accept that shame, as many other emotions, can be both functional and dysfunctional (Cibich et al. 2016).

According to Cibich et al. (2016), emotions are not dysfunctional in nature, but if experienced intensively, frequently or inappropriately relative to the situation, they can become so. A functional interpretation of emotions posits that they serve purposes that have historically assisted survival. These theories also argue that shame is not an ‘ugly’ emotion, but rather that it evolved to protect individuals' social bonds and status. This goal is served by working as an alarm bell for threats to social belongingness. Following this definition, shamelessness is considered a potentially problematic state. For example, a child who bullies others and does not feel ashamed for his or her own bullying behaviour may blame others and become more aggressive. In contrast, a child who bullies others and does feel ashamed can use the feeling of shame as a signal that he or she needs to change that behaviour and adapt a more prosocial behaviour.

Recent research has explored the adaptive functions of shame. In the interpersonal context, De Hooge et al. (2008) have shown that situational shame can motivate prosocial behaviour and serve as a commitment device when it is directly relevant to the current goal (endogenous shame), but not when it is unrelated to it (exogenous shame). Furthermore, they have observed that shame can activate an approach motivation when it is perceived as feasible and not overly risky to repair and maintain a positive self‐image. In situations when an individual is supposed to feel shame, it is likely that shame plays an adaptive role, helping the individual to face the situation (e.g., when a person transgresses or after cheating on a partner). However, when shame is not supposed to be felt (e.g., in situations of physical or mental disability), it is more likely to play a maladaptive role (Dempsey 2017).

### 1.2 Defining and Conceptualizing Shame

After stressing the relevance of shame for psychology and psychopathology, arguing that it can take functional and dysfunctional forms, in this section, we will review some of the main definitions of shame that have appeared in the relevant literature. According to Goss and Allan (2009), shame is a multifaceted, self‐conscious emotion that involves affective, social, cognitive, behavioural and psychological components. Several scholars recognize that one fundamental aspect of shame is the negative social evaluation—sustaining the view that shame is elicited by the public revelation of a characterological flaw or failure. In seminal writings, Lewis (1997) defined shame as an ‘unpleasant and hostile’ condition, attributing to it not only a public failure but also a private nature. In his view, shame is not the result of specific circumstances, but rather it arises from the interpretation and evaluation that each person privately makes of a given situation. Kohut (1971) theorized shame as the collapse of self‐esteem in response to parental failures. According to Kohut (1971), shame can be considered a narcissistic disturbance, one linked to the ‘grandiose self’ or ‘idealized other’ positions. Optimal development requires the child to experience a degree of admiration and empathy from others or to form a positive, idealized attachment to a parental figure. In such instances, the inevitable setbacks and disappointments are less likely to compromise the child's self‐esteem.

Similarly, Scheff (2003) proposed that shame is the principal social emotion, originating from all those situations where a social bond is perceived to be under threat, ascribing to shame both a self dimension and a societal dimension. In line with this, several scholars define shame as a public emotion. Tangney et al. (2007) went as far as to define shame as ‘the more public of emotions’, composed of two elements. The first element is related to the self, and the second is based on the perception of being negatively seen by others—i.e., the social factor.

Another influential perspective on shame (Gilbert 2000, 2007; Matos et al. 2013) based on social rank theory posits that the perceptions of one's social status as superior or inferior to others influence emotions. When one perceives the self as inferior to others, a common outcome is submissive behaviour. In this context, shame is a reflection of defensive submissive strategies when a person perceives the self in an unwanted low‐status position. Notably, perceptions of being in a low‐status position may stem from being passively ignored or from overt rejection; in both cases, shame acts as a reminder we ‘live in the minds of others’ as someone who is unattractive and undesirable (Gilbert 2007). In a similar vein, Dickerson et al. (2004) characterized shame as a self‐conscious emotion whose core causes lie in negative judgement of self as inferior and inadequate. Individuals that are experiencing shame show behaviours linked to the desire to ‘hide’, ‘escape’, ‘disappear from view’ and ‘shrink into the floor’—coherent with feelings of submission and withdrawal. The social nature of shame is also evident in Elison's (2005; see also Elison et al. 2014) work, which stresses the central role of interpersonal devaluation for shame wherein shame is characterized to function in a similar fashion than pain.

In a recent review by Murphy and Kiffin‐Petersen (2017), the definition of shame as a moral emotion was connected to the interests and well‐being of the whole society and not only of ‘a judge or an agent’, thus emphasizing the public side of this emotion. From this point of view, because fostering relationships and interpersonal bonds is a fundamental human motivation, it is clear that shame—as an emotion that is embedded within a relational context—can be a painful and uncomfortable emotion. Moral norms and expectations constructed by society are internalized by people, and this influences the degree of shame that can be experienced (Murphy and Kiffin‐Petersen 2017). However, as it is evident from the foregoing characterization of shame as a multifaceted construct, different perspectives can be embraced to conceptualize and measure it. Two such perspectives differentiate internal versus external shame and trait versus state shame.

#### 1.2.1 Internal and External Shame

An important categorization found in the literature on shame differentiates internal and external shame. Gilbert (1997) was the first to describe the existence of two dimensions, an internal dimension and an external one. The first refers to an evaluation of the self as inadequate, the second to the fear that others will perceive oneself as morally defective. Taylor (2015) defined internal shame as the one occurring when an individual is led to self‐deprecation and a perception of oneself as weak and undignified, stemming from the feeling of having fallen short of one's own values. Critically, even internal shame may have a social nature, when it stems from devaluation from an imagined other, recognized or simply internalized from childhood experiences (Elison 2005).

According to Goss and Allan (2009), internal shame is associated with self‐hatred and strong self‐criticism. On the other hand, external shame refers to the fear and worry of being evaluated negatively by other people (Angulo et al. 2019). This kind of shame is proportional to the importance given to the opinion of others (Proeve and Howells 2002), as the attention is mainly directed outward and focused on guessing what is going on in another person's mind (Goss and Allan 2009). External shame is also associated with social anxiety and worries on how others will consider and judge any action taken—leading to the ashamed self feeling inferior, imperfect, disgusting, weak, inadequate and diminished (Wilson et al. 2006). Rather than being separate entities, internal and external shame tend to be correlated with one another; when an individual perceives himself or herself as inadequate (internal shame), it is more plausible that he or she thinks that others will share the same perception (external shame) (Shepard and Rabinowitz 2013).

#### 1.2.2 State Shame, Trait Shame and Shame‐Proneness

Shame can be experienced in two different ways: as a momentary and acute feeling (state shame) or as a penetrating and introjected feeling (trait shame; Tangney et al. 2007). Shame research has focused more on conceptualizing shame as a trait or disposition, rather than focusing on state‐based shame responses that are specific to a certain trigger. Indeed, what is alternatively termed dispositional or trait shame is characteristic of a person and does not refer to the various situations in which it might occur (Leeming and Boyle 2004). Leeming and Boyle (2004) defined trait shame as a general disposition and, as such, not expected to show different degrees of likelihood in different contexts. While occasional shame (state shame) can be functional and also serve social goals, a dispositional proneness to shame is characterized by a too frequent and too pervasive experience of shame, hence being associated with more maladaptive outcomes (Szentágotai‐Tătar et al. 2020). The literature on shame‐proneness has grown partly separate from trait or dispositional shame, positing that placing shame‐proneness exclusively in the personal (as opposed to social or interpersonal) realm is challenging, given that boundaries between the personal and social world are definitely blurry (Tangney et al. 2007). For instance, if someone grows up in hostile environments, proneness to experiencing shame may be inaccurately attributed to a trait disposition rather than to the environment. In other words, referring to shame as a trait does not imply that it is based on an individual's predisposition. Individuals that are shame‐prone are more likely to externalize blame and experience episodes of intense anger, expressed sometimes in destructive ways which can lead to direct aggression (physical or verbal), indirect aggression, displaced aggression or self‐directed aggression. Alternatively, anger can also be suppressed and remain unexpressed (Tangney et al. 2007). Tangney et al. (2007) argued that shame‐prone individuals are also more likely to experience anticipatory shame, irrespective of the anticipated event. This is directly linked to the likelihood of experiencing felt shame in a range of different circumstances (social contexts or as a reaction to perceived low performance).

### 1.3 Measuring Shame

According to Lear et al. (2022), the multifaceted nature of shame has necessitated diverse methodological approaches to its assessment. Recent research suggests that multidimensional measures, which differentiate between shame‐related cognitions, affect and behaviours or focus on specific domains (e.g., body‐focused shame), are more effective than generalized unidimensional measures (Velotti et al. 2017). Shame‐proneness has been primarily assessed using hypothetical scenarios to gauge an individual's susceptibility to shame responses in various situations, while trait shame has been predominantly measured through self‐report questionnaires that evaluate the frequency and intensity of reported shame experiences.

Robins and Noftle (2006) provided an overview of self‐report measures of shame and other self‐conscious emotions where they distinguished between situation‐based scales, scenario‐based scales, statement‐based scales and adjective‐based scales. In the scenario‐based tests, the person being tested reads a series of situations specifically constructed to evoke certain emotions and assigns a value to the extent to which he or she feels inclined to experience them. The scenario‐based test measuring shame that is most frequently cited in the existing literature is the Test of Self‐Conscious Affect (TOSCA) (Tangney et al. 1989). Ferguson et al. (2007) argued that this test, its predecessors (e.g., SCAAI, Tangney et al. 1989) and its successors (TOSCA‐3, Tangney et al. 2000; TOSCA‐4, Tangney et al. 2008) are the most widely used tools. Although an in‐depth analysis of the TOSCA would go beyond the scope of the study, it is worth emphasizing that it has received some criticisms. Most notably, some authors (e.g., Ferguson et al. 2007; Fontaine et al. 2004) have noted that (a) TOSCA might represent shame in an overly maladaptive fashion (i.e., intensely negative thoughts and emotions) while overlooking its adaptive function, in comparison to its operationalization of guilt and (b) TOSCA might be overdiscriminating between shame and guilt or include item‐level biases that could contribute to such overdiscrimination.

The most common trait measures include the Personal Feelings Questionnaire‐2 (PFQ‐2) (Harder and Lewis 1987), the Internalized Shame Scale (ISS) (Cook 1987), the Adapted Shame and Guilt Scale (ASGS) (Harder and Zalma 1990), the Experience of Shame Scale (ESS) (Andrews et al. 2002), the Experiential Shame Scale (ELSS) (Turner and Waugh 2001), the Other as Shamer (OAS) scale (Goss et al. 1994) and the External and Internal Shame Scale (EISS) (Ferreira et al. 2020). Wordlist surveys have been considered to be more internally coherent than scenario‐based ones. The latter have been criticized due to their limited spectrum of shame‐inducing scenarios, often including situations that are less appropriate and seldom experienced by people with mental issues—e.g., working environment (Rüsch et al. 2007).

### 1.4 Defining and Conceptualizing Shame Regulation

In addition to considerations on the experience of shame, critical to the understanding of adaptive and maladaptive correlates of shame is consideration of how individuals respond to it, that is, shame regulation or shame coping. For instance, shame is more likely to be problematic when the individual tries to avoid it, leaving shame essentially unresolved. Such an avoidance response is considered more likely when the cause of shame is perceived as irreparable (Cibich et al. 2016).

Self‐conscious emotions, such as shame, play an important role in self‐regulation: They help individuals become aware of their social mistakes and, consequently, modify their behaviours (Beer and Keltner 2004). There are multiple ways in which individuals can regulate or cope with shame feelings. According to Nathanson (1992), individuals can recognize their shame and consequently find ways to cope with it or may wilfully or unconsciously avoid it—leading to the so‐called ‘unacknowledged shame’. Nathanson (1992) went further by developing the ‘compass of shame’ model, grouping four categories of maladaptive shame‐coping strategies: withdrawal, avoidance, attack on self and attack on others. All of these are aimed at defending the self from the painful experience of shame, can be endorsed to different degrees and depend on the context. While attack on self involves internalization, self‐directed anger and even disgust, attack on others, on the contrary, is linked to externalization of blame and anger and even aggression. Withdrawal represents a combination of internalization and desire to reduce social exposure. Withdrawal is considered to be the prototypical reaction to shame, as shame naturally leads to a loss of muscle tone in the neck and back, driving a tendency to avoid eye contact by turning eyes and head downcast (Hahn 2001). Finally, avoidance is usually associated with minimization or denial of shame—attempting to reduce social exposure or to keep shame from conscious awareness—and is the only strategy that can be characterized by a lack of conscious experience of shame (Elison et al. 2014; Garofalo and Velotti 2021). Of note, recent theoretical and empirical work (e.g., Capinha et al. 2021; Garofalo and Velotti 2021) has extended the compass of shame model to include a fifth dimension reflecting adaptive coping to shame, suggesting that adaptive coping is not limited to not endorsing maladaptive coping strategies but also include actively adopting adaptive strategies such as seeking social support.

Elison et al. (2014) examined Nathanson's (1992) model in correspondence to evolutionary‐based fight‐or‐flight responses, stressing the transdiagnostic relevance of maladaptive shame coping for various forms of psychopathology spanning internalizing and externalizing spectra. Notably, the four shame‐coping strategies described above are also used in an anticipatory fashion, to avoid the potential experience of shame; these kinds of preventive strategies are not strongly linked to shame‐proneness but rather to shame aversion (Elison et al. 2014). Schoenleber and Berenbaum (2012) described various forms of preventive coping strategies for shame: achievement sabotage, dependence, fantasy, perfectionism and interpersonal avoidance. Achievement sabotage is the attitude to compromise one's performance as in, for example, the procrastination process that can help to avoid the possibility of shame by postponing actions further and further in time. Dependence consists of delegating one's duties to another person to avoid the onset of shame that could have been caused by one's actions. Through fantasy, people can portray themselves as having attractive characteristics that can lead them to desired consequences, shifting attention away from the shortcomings they actually think they have. Perfectionism entails aiming for unrealistic levels of results; individuals engaging in self‐promotion, non‐disclosure and/or non‐exposure will try to find a means of mitigating the anticipated psychological distress associated with the exposure of personal imperfections. Finally, interpersonal avoidance refers to behaviours aimed at avoiding social interactions that could prove to be a source of shame, somewhat akin to Nathanson's (1992) withdrawal, but enacted *before* a potentially shameful situation rather than *after* a shame‐eliciting situation.

### 1.5 The Present Study

The diverging and, at times, inconsistent ways to conceptualize and operationalize shame and shame regulation increase the risk of jingle–jangle fallacies wherein two different constructs are given the same name (jingle fallacy) or when the same constructs are given different names (jangle fallacy). These instances create obstacles to scientific progress as they make integration across studies more difficult. Therefore, given the disparate definitions and measurements present in the literature, two problems arise: The first concerns integration among prevalent conceptualizations and measures in the literature on shame and shame regulation *across* studies. The second concerns gauging the level of consistency between conceptualizations and methods of operationalizations of shame and shame regulation—that is, whether there is coherence *within* studies between how shame and shame regulation are defined and measured. Given the vastness of the literature on the topic, review studies that synthesize primary literature have accumulated in the past few decades. Therefore, we opted to conduct an umbrella review of recent synthesis studies to encompass a wider range of literature which has already undergone screening and integration using systematic review and/or meta‐analytic methodology. This methodological approach is beneficial when the goal is to draw an overall integrated view regarding a broad topic by synthesizing review studies that each cover a large number of published studies across different populations (Choi and Kang 2023). The focus on recent review studies (published between 2018 and 2023; see Section 2) was motivated by a desire to gauge contemporary trends in the shame literature based on the most recent reviews. This allowed the coverage of earlier empirical findings as they were synthesized in the reviews included in the present umbrella review, while keeping the volume of the literature to screen manageable.

Against the conceptual and empirical background reviewed above, the aims of the present review were to systematically review conceptualizations and measurements of shame and shame regulation. Specifically, we examined whether the constructs of shame and shame regulation have been defined mostly within a unidimensional or a multidimensional framework, whether methods of operationalizations of shame and shame regulation have been based on unidimensional or multidimensional measures and whether there is consistency within studies on how shame and shame regulation are conceptualized and measured. A further goal of this review was to provide an integrated perspective on consensual definitions of shame experience and regulation, as well as an overview of the measures most commonly used to assess them. Finally, a secondary goal of the review was to provide a meta‐synthesis of the main findings to emerge from the included reviews, with an emphasis on the links that shame and shame regulation have with psychopathology.

## 2 Method

**FIGURE 1:**
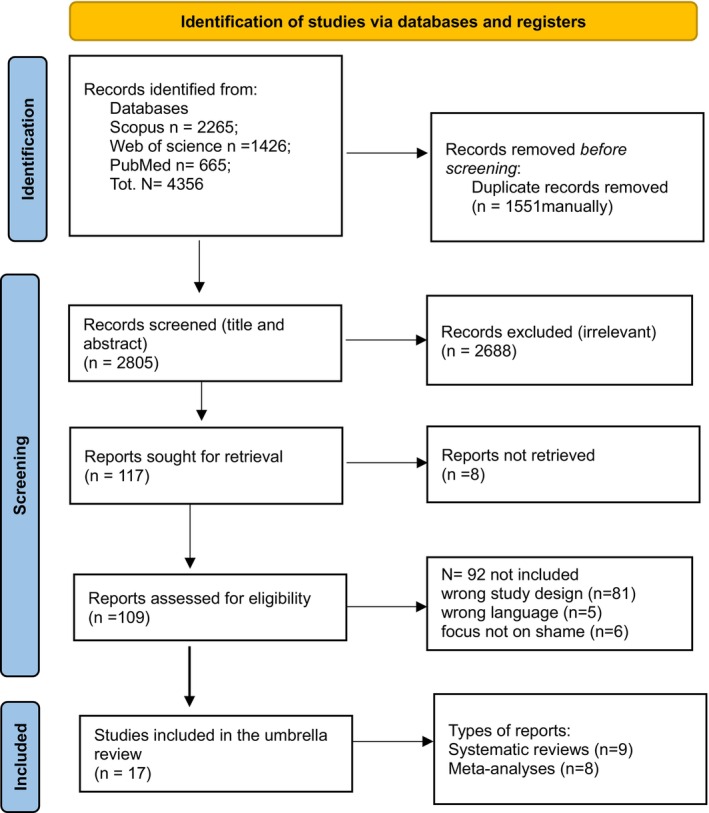
PRISMA flow chart.

Relevant studies were identified by conducting a literature search through the following databases: Scopus, Web of Science and PubMed. The literature search was conducted on February 24, 2024. The selection process considered the most recent articles on the topic, focusing on a 6‐year period that included publications from 2018 to 2023. The following key search terms were used: ‘shame AND (review OR meta‐analysis OR systematic OR scoping OR synthesis)’. The search strategy yielded 4356 articles. Out of these, 1551 duplicates were manually removed, and a total of 2805 articles were screened for relevance based on their titles and abstracts. A number of 2688 articles were excluded due to their irrelevance. One hundred seventeen reports were searched for full text retrieval, but eight of them could not be found. At this point, 109 articles were eligible for screening based on the following criteria: main focus on the topic of shame (i.e., excluding studies that have included shame as a corollary to another main theme under investigation); only systematic reviews and meta‐analyses; only publications in English or Italian language. Eighty‐one articles were excluded due to using the wrong study design; five articles were not included because they were not available in English, and six articles were excluded due to their lack of focus on shame. Eventually, a total of 17 studies were included in this review (see Figure 1 for the PRISMA flow chart detailing the screening process).

After the records selection was completed, the following data were extracted from each article included in the umbrella review: title and year of publication of the study; the authors and their affiliations; the journal in which they were published; the objectives of the studies; shame conceptualization (unidimensional or multidimensional); shame regulation and coping strategies; shame measurement tools; and main findings. A quality assessment was conducted for all reviews using a 17‐item checklist (Robinson et al. 2020). Data extraction for 30% of the records was independently repeated by a second author, and no discrepancies emerged.

## 3 Results

Seventeen review studies (nine systematic reviews and eight meta‐analyses) published between 2018 and 2023 were included in this umbrella review. A summary of these studies is presented in Table 1. In this section, the superscript numbers included in square brackets refer to records listed in Table 1, and we use the term ‘studies’ to refer to both systematic reviews and meta‐analyses. Overall, a total of 748 samples were included and approximately 166,172 participants were reached. A total of three studies were published in the *Clinical Psychology Review* journal [^2,5,16^]; two studies came from the *Journal Psychology and Psychotherapy: Theory, Research and Practice* [^12,6^]; two studies were in the *Journal of Traumatic Stress* [^1,3^]. Based on the lead author's main affiliation, the majority of studies were published from the United States (35%), followed by Australia (23%), the United Kingdom (18%), Romania (12%) and Indonesia and Ireland (6% each). Overall, the quality assessment of the study showed that 14 of the studies were of high quality, with only one study being of low quality. Table 1 shows an overview of all included studies.

**TABLE 1:** Overview of studies included in the umbrella review.

No.	Title and year of publication	Authors	Journal	Aim	Shame conceptualization and regulation	Measure used for shame	Main findings	Quality assessment
1	‘A Meta‐Analysis of the Association Between Shame and Dissociation’ (2022)	Rudy et al. (USA)	*Journal of Traumatic Stress* (USA)	To enquire on the association between shame and dissociation	**Unidimensional construct** Shame can be divided into state and trait. *Shame regulation*: Shame usually leads to avoidance of or withdrawal from social risk; dissociation is a mechanism for mitigating shame.	PFQ‐2; SSGS; ESS; ISS; HTQ; PANAS; P; SAQ/SPQ; INTERVIEW	‐ There is a significant moderate relationship between shame and dissociation, but no explicit clinical moderators emerged.	High quality
2	‘An Association of the Relationship Between Shame, Guilt and Self‐Harm: A Systematic Review and Meta‐Analysis’ (2019)	Sheehy et al. (UK)	*Clinical Psychology Review* (USA)	To investigate the relationship between self‐harm and shame and guilt	**Multidimensional construct** Shame can be external and internal. Shame may result from different factors of the self: character, behaviour or body. *Shame regulation*: Rumination, submission, avoidance and self‐concealment are common strategies for coping with shame.	PFQ‐2; ISS; OBCS‐Y; OBCS; ESS; TOSCA‐3; SVQ; Shame and Guilt scale; OAS; Test of Self‐Conscious Affect for Socially Deviant populations (TOSCA‐SD; emotional traits assessed via NEO Personality Inventory, revised (NEO‐PI‐R); TOSCA‐4; TOSCA; OAS‐2, Portuguese version	Shame and self‐harm are positively linked. Shame had a positive association with both suicidal behaviour and self‐harm.	High quality
3	‘Association Between Shame and Posttraumatic Stress Disorder: A Meta‐Analysis’ (2019)	López‐Castro et al. (USA)	*Journal of Traumatic Stress* (USA)	To examine the association between shame and PTSD symptoms in trauma‐exposed individuals To clarify the moderators in the relation between shame and PTSD	**Multidimensional construct** This covers behavioural, characterological and bodily aspects. *Shame regulation*: Shame‐triggered distress might manifest as self‐harm, aggression, withdrawal and isolation.	PFQ‐2; TOSCA; ISS; the Abuse‐Specific Shame Questionnaire	There is a positive association between self‐reported shame and PTSD symptoms. There is evidence that shame is important to PTSD symptomatology.	High quality
4	‘A Systematic Review of Self‐Report Measures of Generalized Shame’ (2022)	Lear et al. (USA)	*Journal of Clinical Psychology* (USA)	‐ To recognize and assess the measurement characteristic of self‐report measures of generalized shame	The **multidimensional approach** is more effective in capturing the complexity of the construct: internalized (how I see myself) vs. externalized (how I think other see me) *Shame regulation*: Shame leads to appeasement or withdrawal to avoid social conflict.	ASGS; EISS; ESS ELSS; ELSS‐10; ISS; ISS‐35; OAS; OAS‐2; PFQ PFQ‐2; PFQ‐2 Brief; SCAAI; Shame Inventory; TOSCA; TOSCA‐3; TOSCA‐3 SF; TOSCA‐SD	Generalized shame measures are limited in scope and quality. Scenario‐based measures are less suitable for describing life events than experience‐based measures.	High quality
5	‘Examining the Relationship Between Shame and Social Anxiety Disorder: A Systematic Review’ (2021)	Swee et al. (USA)	*Clinical Psychology Review* (USA)	‐ To analyse the relationship between shame and social anxiety	**Multidimensional construct** Shame can be internal (negative evaluation of the self by the self) and external (negative public perception). Shame is composed of different dimensions: cognitive, character and psychological. *Shame regulation*: Shame and social anxiety share avoidance, escape, withdrawal and submissive behaviours.	ESS; OAS; BSGQ‐shame; PACR‐SS; disconnection/rejection schemas (e.g., shame): YSQ‐3; TOSCA‐3; PFQ‐2‐shame; PACR; IAT; OBCS; DCQ‐shame; TOSCA‐shame; ADCQ; PBIQ; YSQ‐S; ISS; GROSCA; SSGS‐shame; ASGS; TOSCA‐C‐shame; SCEMAS; SQ; ExpSS; PANAS‐shame; ShARQ; TOSCA‐3, short version; PSDQ	There is a stable positive association between shame and SA. This link could be present in different cultures and clinical situations. Early adverse experiences are positively linked with both shame and SA. There is a bidirectional positive association between shame and significant cognitive–behavioural processes in SAD.	Reasonable quality
6	‘Experiences of Shame and Guilt in Anorexia and Bulimia Nervosa: A Systematic Review’ (2018)	Blythinet et al. (UK)	*Psychology and Psychotherapy: Theory, Research and Practice* (UK)	‐ To assess the link between shame, guilt, and AN and BN	**Multidimensional construct** There are differences between internal and external shame. *Shame regulation*: Shame coping mechanisms include trying to control weight and appearance.	PANAS; Body Shame Questionnaire; OAS, PFQ; ESS; TOSCA; OAS (Portuguese version); ESS modified; DES (German version); DES‐IV; BSS	Support for the role of shame in AN and BN presentations is provided; shame is positively associated with the gravity of symptoms. Shame contributes to the onset of dysfunctional eating behaviours (e.g., binging or purging).	High quality
7	‘Exploring the Effectiveness of Mindfulness‐Based and Third Wave Interventions in Addressing Self‐Stigma, Shame and Their Impacts on Psychosocial Functioning: A Systematic Review’ (2022)	Stynes et al. (IE)	*Journal of Contextual Behavioral Science* (USA)	To specify the effectiveness of mindfulness‐based and third‐wave interventions on self‐stigma and shame The effect of the interventions on psychosocial functioning areas	**Multidimensional construct** There can be external shame and internal shame: Others rate myself negatively; and internal shame leads to a negative self‐assessment. *Shame regulation*: Avoiding eye contact and withdrawal are strategies to prevent exclusion and rejection.	**E**SS; ISMIS; ISS; OBCS; OAS; PANAS; PANAS‐X; PFQ‐2; SCES; SHAME; SSGS; SSMIS; TOSCA‐3; TOSCA‐4; VAS; WSSQ	MBTW can be useful in helping individuals cope with self‐stigma and shame. Self‐compassion intervention or ACT can be beneficial in dealing with self‐stigma and shame. MBTW interventions are helpful in promoting well‐being and psychological health.	High quality
8	‘Interventions to Reduce Shame: A Systematic Review’ (2020)	Goffnett et al. (USA)	*Journal of Behavioral and Cognitive Therapy* (NL)	To investigate interventions to reduce shame To sum up the instruments used in its measurement	**Unidimensional construct** Shame involves a negative cognitive evaluation of the self. Shame makes people feel disappointed, undeserving, being a failure and being not good enough. *Shame regulation*: The pain of shame drives people to withdrawal and to a cycle of dysfunction.	ISS; PFQ‐2; TOSCA; ELSS; Human Immunodeficiency Virus (HIV) Shame subscale from the Social Judgment Stigma Scale (SJSS); OBCS	‐ Of 37 evidence‐based interventions, 33 were successful in mitigating post‐intervention shame.	High quality
9	‘Shame and Binge Eating Pathology: A Systematic Review’ (2022)	O'Loghlen et al. (AU)	*Clinical Psychology & Psychotherapy* (UK)	To explain what types of shame are associated with binge eating symptoms To analyse factors that anticipate or maintain the relationship between shame and BE	**Multidimensional construct** Shame can be internal and external shame, involving body shame and binge eating–related shame. *Shame regulation*: Binge eating episodes aim to mitigate negative emotions.	Shame was measured using several types of instruments, but they are not provided.	Internal and external shame are positively associated with binge eating behaviour. Body shame can predispose to binge eating behaviours. Shame about binge eating may maintain BE symptoms.	High quality
10	‘Shame and Eating Disorders Symptoms: A Meta‐Analysis’ (2021)	Nechita et al. (RO)	*International Journal of Eating Disorders* (UK)	‐ To investigate the association between shame and disordered eating	**Multidimensional construct** Shame incorporates various forms of negative self‐evaluations such as internal and external. Shame is a state emotion or shame‐proneness. *Shame regulation*: Binge eating and attempts to control food intake regulate shame, but BE can perpetuate shame, and this creates a vicious circle.	BIGSS; BRSS; BISS‐A; BSQ; BSS; DAS‐shame; EBSS; EISS; ES‐ESS; ESS; IBSS, ISS; OAS; OBC, OBC‐Youth, PBSS; PFQ‐2; SSGS; GES, The Shame and Guilt Eating Scale; TOSCA; WEB‐ SG‐SH, The Weight‐ and Body‐Related Shame and Guilt Scale, Shame subscale WFES, Weight‐Focused External Shame Scale	The form of shame associated with ED symptoms could be body shame and shame related to eating. Effect size is medium or large in the link to shame or ED, respectively.	High quality
11	‘Shame and Guilt in the Postnatal Period: A Systematic Review’ (2020)	Caldwell et al. (AU)	*Journal of Reproductive and Infant Psychology* (UK)	‐ To research the role of shame and guilt to postnatal psychological symptom	**Multidimensional construct** Internal shame is when the individual criticizes and accuses himself. External shame is a negative emotion that comes from thinking that others perceive the self as inadequate. *Shame regulation*: Regulation strategies include avoidance and aggression.	TOSCA‐3; GI; ERSGS	There is a positive association of shame and stress (acute, parental and posttraumatic) and postnatal depression in mothers. Stress condition (acute, parental and posttraumatic), anxiety and depression are present in fathers.	Low quality
12	‘Shame and the Psychosis Continuum: A Systematic Review of the Literature’ (2020)	Carden et al. (UK)	*Psychology and Psychotherapy: Theory, Research and Practice* (UK)	‐ To study the relationship between shame and the psychosis continuum	**Unidimensional construct** Shame is a trait or a dispositional proneness. There is an explicit reference to external shame. *Shame regulation*: Regulation involves ‘the urge to escape and withdraw’.	ISS, TOSCA‐2; ESS; OAS; TOSCA; OAS Portuguese; ESS Portuguese; ISS Portuguese; CES Portuguese; ASGS; PFQ‐2; DES; ERSQ‐ES	There is a positive link between shame and psychotic/psychotic‐like experiences (e.g., paranoia). Shame may play a significant role in explaining psychosis/psychosis‐like experiences.	High quality
13	‘Shame and Self‐Esteem: A Meta‐Analysis’ (2021)	Budiarto et al. (ID)	*Europe's Journal of Psychology* (RO)	‐ To investigate the relationship between shame and self‐esteem	**Multidimensional construct** Shame is present in many areas of life such as behavioural or physical ones. A multidimensional way is useful to differentiate the various areas in which shame is involved. *Shame regulation*: This is not provided.	No provided shame measures	‐ There is a negative correlation between shame and self‐esteem.	High quality
14	‘The Association Between OCD and Shame: A Systematic Review and Meta‐Analysis’ (2022)	Laving et al. (AU)	*The British Journal of Clinical Psychology* (UK)	‐ To analyse the association of shame with OCD and unacceptable thoughts	**Unidimensional construct** There is a distinction between trait and state shame. *Shame regulation*: Regulation includes social withdrawal, avoidance, delayed treatment search and reluctance to share details of the symptoms.	Young Schema Questionnaire ‐Shame/Defectiveness subscale (YSQ); TOSCA; SSGS	Shame and OCD symptom are positively associated. Shame is a common experience for individuals diagnosed with OCD. Shame is an obstacle to getting help.	High quality
15	‘Shame‐Proneness, Guilt‐Proneness and Anxiety Symptoms: A Meta‐Analysis’ (2018)	Cȃndea et al. (RO)	*Journal of Anxiety Disorders* (USA)	‐ To summarize the association between shame, guilt and anxiety symptoms	**Multidimensional construct** Shame is not a homogeneous construct: It can be internal and external shame. *Shame regulation*: Shame leads to withdrawal when it is impossible to recover the vision of oneself.	TOSCA‐3; ISS; OAS; IAT‐Shame; PFQ‐2; CSGS‐A; DCQ; ESS; SSGS; ARS; ADCQ; ARBQ; ASGS; CAPS‐CA; OAS‐2; SCEM AS; BSGQ‐C; VAS; A‐SSS; SHAME; PANAS; SCAAI TOSCA; A‐3‐SF; IPARS	Shame and guilt are positively linked with anxiety symptoms (medium effect size). Shame and partial shame had positive correlations with both state and trait anxiety (medium effect sizes). Shame was not connected with panic symptoms.	Reasonable quality
16	‘Substance Use and Shame: A Systematic and Meta‐Analytic Review’ (2019)	Luoma et al. (USA)	*Clinical Psychology Review* (USA)	‐ To sum up the associations between shame and substance use or substance use–related problems	**Unidimensional construct** Shame is understood as an adaptive and maladaptive emotion. Shame‐proneness (trait) is compared with shame lived experience (state). *Shame regulation*: Regulation involved avoidance and withdrawal to defend the weak and unpleasant self; substance use is a form of shame avoidance.	NEO‐PI‐R (shame factor); YSQ‐L3; TOSCA, TOSCA‐GFS; TOSCA‐SD‐GFS; PANAS; OBCS‐BS; SSGS (modified); CoSS; PFQ‐2; ISS; State shame; IS; ashamed mood (daily report); AAII; nonverbal shame, SI; HIV‐related shame; YSQ‐S3; TOSCA‐C‐GFS; TAS; IPARS	There is no consistent relation between substance use and shame. Among substance users, there is a relation between shame and substance use–related difficulties.	High quality
17	‘Was it me? The Role of Attributions and Shame in Post‐Traumatic Stress Disorder (PTSD): A Systematic Review’ (2023)	Seah et al. (AU)	*Trends in Psychology* (BR)	To review the relationship between attributions, shame and PTSD. To verify if shame would explain the relationship between attributions and PTSD	**Unidimensional construct** Shame is a state or trait. There is a failure in meeting internal or external criteria. *Shame regulation*: Withdrawal is a craving to hide the self.	Abuse‐Specific Shame Measure; The Trauma‐Related Shame Inventory (TRSI)	There is a positive relation between shame and PTSD. Attributions and trauma‐specific or attributional style are positively related to shame and PTSD symptoms.	High quality

### 3.1 Conceptualization of Shame and Shame Regulation

The majority of studies (64.7%) that have been reviewed agree with defining shame as a multidimensional construct. In particular, most articles (53%) proposed shame as a construct that can be categorized according to two dimensions that in literature are identified as internal and external shame [^2,4,5,6,7,9,10,11,15^]. Six studies (41.2%) [^1,8,12,14,16,17^] stressed the distinction between trait and state shame but considered them as unidimensional constructs. Four other studies (23.5%) [^2,3,10,13^] embraced a multidimensional definition of shame as consisting of disparate aspects: character, bodily components, physical components and various aspects of the self.

Conceptualizations of shame regulation were also examined, and most of them were directly or indirectly based on Nathanson's (1992) compass of shame model. In most of the studies included, withdrawal was the shame‐coping strategy most often investigated (in 11 studies). One study investigated shame regulation through the avoidance of eye contact [^7^] in the context of interpersonal conflict. More generally, seven studies found avoidance to be a coping strategy to avoid social risks. Two studies reported aggression as a strategy [^3,11^] both inwardly and outwardly directed.

Some studies dealing with shame and eating disorder symptoms [^6,9,10^] have inferred that trying to control one's weight and physical appearance is a way to deal with shame [^6^]. Similarly, binge eating episodes have been considered as possible behaviours that mitigate negative emotions, and in addition to this, the control of food intake was considered as a coping strategy for shame. At the same time, shame seemed to be able to perpetuate dysfunctional eating behaviours and the control of nutrition, contributing to establishing a vicious cycle between maladaptive shame regulation and dysfunctional eating behaviours. One study reported that dissociation could reduce the symptoms of shame [^1^], and it has also been hypothesized that substance abuse can be a way to escape shame [^16^].

### 3.2 Measures of Shame and Shame Regulation

The studies examined contain a total of 74 instruments used for measuring shame. It emerged that the most used instrument for measuring shame is the TOSCA. TOSCA has been used in 14 studies [^2,3,4,5,6,7,8,10,11,12,14,15,16^] in its different versions (TOSCA‐2 in one study, TOSCA‐3 in seven studies, TOSCA‐4 in two studies, TOSCA‐SD in three studies and SCAAI TOSCA in one study). The PFQ‐2 has also been widely used, as seen in its usage within 12 papers [^1,2,3,4,5,6,7,8,10,12,15,16^]. Eleven studies [^1,2,3,4,5,7,8,10,12,15,16^] reported using the ISS, and nine used the ESS [^1,2,4,5,6,7,10,12,14^]. Eight studies have employed the OAS [^2,4,5,6,7,10,12,15^]; seven studies, the State Shame and Guilt Scale (SSGS) [^1,5,7,10,14,15,16^]; five studies, the Positive and Negative Affect Scale (PANAS) [^1,6,7,15,16^]; four papers, the ASGS [^4,5,12,15^]; four papers, the Objectified Body Consciousness Scale (OBCS) [^2,5,7,8^]; and three studies the Shame Assessment for Multifarious Expressions of Shame (SHAME) [^4,7,15^]. The other measuring instruments were used in two studies or only one study. Most notably, only two reviews included measures of shame regulation: in one case, the Compass of Shame Scale, and in another case, the less specific Emotion Regulation Skills Questionnaire. For a complete overview of the most used tools in the reviews, see Table 2.

**TABLE 2:** Overview of measures drawn from the included studies, used to assess shame experience (E) or regulation (R) (in alphabetical order).

AAII	Acceptance of an Alcoholic Identity Instrument	E
ADCQ	Adapted Dimensions of Conscience Questionnaire	E
ABRQ	Abuse‐Related Beliefs Questionnaire	E
ARS	Abuse‐Related Shame	E
A‐SSS	Arabic Self‐Shame Scale	E
ASGS	Adapted Shame and Guilt Scale	E
ASSQ	Abuse‐Specific Shame Questionnaire	E
BIGSS	Body Image Guilt and Shame Scale	E
BISS‐A	Body Image Shame Scale for Adolescents	E
BSGQ‐C	Brief Shame and Guilt Questionnaire for Children	E
BSQ	Body Shame Questionnaire	E
CAPS‐CA	Clinician‐Administered PTSD Scale for Children and Adolescents	E
CoSS	Compass of Shame Scale	R
CSGS‐A	The Caring Shame and Guilt Scale, adapted	E
DAS‐SHAME	Differential Affect Scale, Shame subscale	E
DCQ	Dimensions of Conscience Questionnaire	E
EBSS	The Externalized Bodily Shame Scale	E
EISS	External and Internal Shame Scale	E
ELSS	Experiential Shame Scale	E
ERSGS	Event‐Related Shame and Guilt Scale	E
ERSQ‐ES	Emotion Regulation Skills Questionnaire	R
ESS	Experience of Shame Scale	E
GROSCA	Global Ratings of Self‐Conscious Affect Scale	E
HIV‐RELATED SHAME	Human Immunodeficiency Virus Shame subscale	E
HTQ	Harvard Trauma Questionnaire	E
IAT‐SHAME	Implicit Association Test, Shame	E
IES‐R	Impact of Event Scale, revised	E
IPARS	Intimate Partner Aggression‐Related Shame Scale	E
ISS	Internalized Shame Scale	E
ISMIS	Internalized Stigma of Mental Illness Scale	E
NEO‐PI‐R	Neuroticism–Extraversion Openness Personality Inventory Revised	E
OAS	Other as Shamer	E
OAS‐2	Other as Shamer‐2	E
OBCS	Objectified Body Consciousness Scale	E
OBCS‐BS	Objectified Body Consciousness Scale, Body Shame subscale	E
OBCS‐Y	Objectified Body Consciousness Scale, Youth	E
PACR‐SS	Shame Subscale of the Parental Attitudes Toward Children Rearing Scale	E
PANAS	Positive and Negative Affect Scales	E
PANAS‐X	Positive and Negative Affect Scale, expanded version	E
PBIQ	Personal Beliefs About Illness Questionnaire	E
PBSS	Phenomenological Body Shame Scale	E
PFQ‐2	Personal Feelings Questionnaire‐2	E
PSDQ	Parenting Styles and Dimensions Questionnaire	E
SAQ/SPQ	Shame Attributions Questionnaire/Shame Picture Questionnaire	E
SCAAI	The Self‐Conscious Affect and Attribution Inventory	E
SCEMAS	The Self‐Conscious Emotions: Maladaptive and Adaptive Scales	E
SCES	Self‐Conscious Emotion Scale	E
SHAME	Shame Assessment for Multifarious Expressions of Shame	E
ShARQ	Shame‐Aversive Reactions Questionnaire	E
SI	Shame Inventory	E
SGES	The Shame and Guilt Eating Scale	E
SPM	Shame Posture Measure	E
SSGS	State Shame and Guilt Scale	E
SSGS‐modified	State Shame and Guilt Scale, modified	E
SSMIS	Self‐Stigma of Mental Illness Scale, short form	E
SQ	Schema Questionnaire	E
SVQ	Shame Variability Questionnaire	E
TAQ	Trauma Appraisal Questionnaire	E
TOSCA	Test of Self‐Conscious Affect	E
TOSCA‐C	Test of Self‐Conscious Affect for Children	E
TOSCA‐SD	Test of Self‐Conscious Affect for Socially Deviant and Incarcerated Groups	E
TOSCA‐2	Test of Self‐Conscious Affect‐2	E
TOSCA‐3	Test of Self‐Conscious Affect‐3	E
TOSCA‐4	Test of Self‐Conscious Affect‐4	E
TRSI	Trauma Related Shame Inventory	E
VAS	Visual analogue scale	E
WEB‐SG‐SH	The Weight‐ and Body‐Related Shame and Guilt Scale, Shame subscale	E
WFES	Weight‐Focused External Shame Scale	E
WSSQ	Weight Self‐Stigma Questionnaire	E
YSQ	Young Schema Questionnaire	E
YSQ‐L3	Young Schema Questionnaire, long form, third edition, Defectiveness/Shame subscale	E
YSQ‐S	Young Schema Questionnaire, short form	E
YSQ‐S3	Young Schema Questionnaire, short form, third edition, Defectiveness/Shame subscale	E
YSQ‐3	Young Schema Questionnaire‐3	E

### 3.3 Main Study Aims and Findings

All studies had different aims and investigated the relationship between shame and various other constructs. Two studies focused on the association between shame and exposure to trauma, post‐traumatic stress disorder and related symptoms [^3,17^]. These studies found that shame and post‐traumatic stress disorder were strongly associated. Three papers analysed the topic of shame related to eating disorders [^6,9,10^]. These studies showed a relationship between shame and the difficulties in eating behaviours and the fact that shame can represent a risk factor for binge eating behaviours. Body shame and shame related to eating seemed to be the types of shame that are most associated with eating symptoms. Swee et al. (2021) [^5^] and Cândea and Szentagotai‐Tătar (2018) [^15^] both related the construct of shame to the spectrum of anxiety disorders, corroborating an association between these two constructs. Shame was associated with social anxiety, both trait and state anxiety, and other types of anxiety except panic symptoms.

Other studies included in this review had different goals in relation to shame, such as the association between shame and dissociation [^1^]; associations among shame, guilt and self‐harm [^2^]; and the link between shame and psychosis continuum [^12^]. Shame was positively associated with all three of these constructs. One study provided support for the hypothesis that there is a correlation between shame and self‐esteem [^13^]; furthermore, one study reported associations between shame and obsessive–compulsive disorder symptoms [^14^]. Associations were found between shame and postnatal symptoms. In particular, one study argued that shame was associated with stress and depression in mothers and with stress, depression and anxiety in fathers [^11^]. Shame did not seem to be consistently linked to substance use, but among substance users, shame was related to greater substance‐related difficulties [^16^]. One study aimed to review self‐report measures of generalized shame [^4^] such as scenario‐based and experience‐based shame, suggesting that scenario‐based measures may be less ecologically valid to life events than experience‐based measures. Two studies have dealt, in different ways, with interventions that can reduce and address problems related to shame. In particular, it has been found that evidence‐based interventions can reduce shame [^8^] and 33 out of 37 studies have documented the reduction of shame post‐intervention. It has also emerged that mindfulness‐based and third wave cognitive–behavioural interventions can be beneficial in supporting individuals with shame problems and can have a positive impact on people's quality of life [^7^]. All main findings are summarized in Table 1.

## 4 Discussion

The aim of this umbrella review was to analyse the conceptualization of the constructs of shame and shame regulation as well as their measurements through the analysis of systematic reviews and meta‐analyses. The synthesis studies included in the present umbrella review were, by and large, of high quality. Overall, shame emerged as being conceptualized in a multidimensional way in the majority of studies, although a non‐negligible portion of studies simply differentiated between state and trait shame, without defining different dimensions within the construct of shame. Among the multidimensional conceptualizations of shame, the most common were external and internal shame as well as the conceptualization of shame as a construct that includes behavioural, physical and bodily factors. While the distinction of internal vs. external shame is based on Gilbert's (1997) seminal contribution, conceptualizations of shame as an emotion that arises and ‘spans’ diverse aspects such as behaviour, body or character (e.g., López‐Castro et al. 2019) are largely based on Andrews et al.'s (2002) empirical work. Another important distinction, albeit not inherently multidimensional, that emerged from the reviewed studies is the consideration of adaptive and maladaptive manifestations of shame; specifically, the experience of shame has been conceptualized as adaptive and functional for social relationships, but maladaptive when it is experienced too intensely and too frequently (Cibich et al. 2016). Finally, studies alternatively used the terms ‘trait shame’ and ‘shame‐proneness’ interchangeably (Luoma et al. 2019; Nechita et al. 2021) or as indicating slightly different things (Carden et al. 2020), the latter more in line with Tangney et al. (2007) conceptualization of shame‐proneness.

Regarding measurements, one of the most used trait measures for shame is the TOSCA, which was included in 76% of the reviewed studies. In contrast, a wide range of measures were implicated in single reviews (or two), such as the Shame Inventory or the EISS. Although the definition of shame regulation was present in the majority of the studies (see Table 1), the included instruments refer predominantly to the measurement of the experience of shame and not to its regulation, with the exception of the CoSS and ERSQ‐ES in two reviews (for an overview, see Table 2).

One of the goals of this review was to investigate whether there was consistency between the conceptualization and measurement of shame. Taking this analysis into consideration, reflections on the coherence between conceptualization and measurement of the shame construct were made on the basis of definitions and instruments that have been most common. This aim examined whether the review studies included a quantitative synthesis in line with the concepts that were the focus of the review. As previously observed, shame has been found to be a multidimensional construct in the vast majority, so it would be reasonable to expect that its measurement would be concerned with measuring the various dimensions. However, it has been found that, among the most used instruments, the majority have been designed or used for a unidimensional operationalization of the construct. This seems to show that conceptualization and measurement lack coherence to some extent, and this could compromise our ability to integrate findings from different studies and to add nuance to our understanding of the multifarious manifestations of shame experience. Specifically, few scales (e.g., ESS, ELSS, EISS, SHAME) are developed to capture multiple dimensions of shame, but findings using those measures are often synthesized in the form of total scores rather than subscale scores. Most other measures can be reduced to a single‐factor operationalization. Notable across both levels concerning the experience and regulation of shame was a general lack of assessment instruments for adaptive forms of shame and adaptive shame regulation (with the exception of the expanded compass of shame model; Capinha et al. 2021; Elison et al. 2014; Garofalo and Velotti 2021), despite widespread theoretical consensus that shame and its regulation can reflect both adaptive and maladaptive processes. Taken together, these issues can contribute to a fragmentation between how shame is conceptualized and how it is operationalized.

In this regard, Lear et al. (2022) argued that a multidimensional approach to shame has gained ground in recent years, which could guarantee a more nuanced approach to assess shame. Tools that fail to capture the nuances of the construct could hinder research and, by extension, clinical practice. Indeed, the variety of definitions of shame makes it difficult to operationalize and measure it, and different instruments are used depending on the dimension of shame that is being measured. However, combining multiple instruments that tap on unidimensional—yet distinct—conceptualizations could represent an efficient compromise to increase alignment between conceptualization and operationalization (e.g., the ISS and the OAS respectively measure internalized shame and external shame). In line with this, Goffnett et al. (2020) argued that it can be very useful to use both general and context‐specific measures of shame to gain a fuller understanding of the construct and to understand which measures are most sensitive to change after intervention in clinical studies. Finally, it is noteworthy that several measures exist to assess shame in relation to specific problems (e.g., alcohol, abuse), implying that there might be something inherently different in measuring problem‐related shame as opposed to shame broadly construed. This appears to be another area in need of conceptual clarity.

According to the literature, shame and shame regulation have been associated with a variety of psychopathological symptoms (Tangney et al. 2007). Even though the association between shame and psychopathological constructs was not among the main objectives of the present study, it is important to elaborate on the connections that emerged from the reviewed studies. This allowed us to underline the transdiagnostic relevance of shame. In fact, this study has documented that many psychopathological conditions have been associated with shame. Specifically, a large literature is available on the association between shame and PTSD and how shame can fuel suffering in the aftermath of trauma (López‐Castro et al. 2019). Luoma et al. (2019) examined substance abuse, arguing that maladaptive shame may be a factor that encourages alcohol use and promotes the maintenance of this behaviour; specifically, while there was no convincing evidence of the link between overall levels of shame and substance abuse, there was consistent evidence that—among substance abusers—shame is related to worse outcomes. In addition to this, evidence has been provided regarding the relationship with other disorders such as eating disorders. For example, binge eating has been associated with body shame and feelings of inadequacy towards the body (O'Loghlen et al. 2022). Beyond this, studies that have analysed the relationship between shame and anxiety disorders have supported a strong correlation between shame and symptoms of social anxiety specifically (Swee et al. 2021; Cândea and Szentagotai‐Tătar 2018), but also with anxiety, stress and depression in parents after birth (Caldwell et al. 2021). Finally, one review showed that shame can contribute to the initiation and maintenance of psychosis (Carden et al. 2020).

Regarding the regulation of shame, the role of withdrawal and avoidance as a coping strategy to deal with shame has been emphasized, in line with Nathanson's (1992) compass of shame model. Moreover, several studies have interpreted some forms of symptomatology as maladaptive strategies to regulate physical shame, suggesting that maladaptive eating behaviours can be, on the one hand, a shame regulation strategy but that, on the other hand, shame can cause the persistence of these behaviours. Binge eating has also been seen as a way of regulating shame (Nechita et al. 2021) along with attempts to control weight and body image (Blythin et al. 2020). In people with OCD, difficulties in regulating emotions, shame in particular, can lead to difficulties in seeking medical treatment and in explaining the symptoms they suffer from due to the shame caused by unacceptable thoughts (Laving et al. 2023). Finally, other symptoms identified as maladaptive mechanisms to regulate shame are dissociation (Rudy et al. 2022) and self‐harm (Sheehy et al. 2019). Overall, limited research appeared focused on synthesizing the evidence linking shame and shame regulation with externalizing forms of psychopathology.

### 4.1 Limitations and Future Research

The present findings should be interpreted in light of the study's limitations. First, the selection criteria led to the inclusion of samples that almost entirely referred to an adult population; hence, our review cannot directly speak for the conceptualization and measurement of shame and shame regulation in children and adolescents. Second, the author's nationality for the included studies, in 16 cases out of 17, was Western. Hence, it was not possible to explore cultural differences. Finally, our focus on review studies published in the 2018–2023 time span represented a contemporary snapshot of shame conceptualizations and measurement but did not allow us to describe scholarly trends over longer periods of time.

### 4.2 Conclusions

As Scheff (2003) put it, shame may be the ‘master emotion of everyday life’, but nevertheless, in most modern societies, it remains a hidden emotion because, too often, it is still considered a taboo (Scheff 2014; Scheff and Mateo 2016). The present umbrella review provided an integration of concepts and methods to study shame based on a critical review of recent synthesis studies (i.e., systematic reviews and meta‐analyses). Despite widespread consensus in theorizing shame and its regulation as complex, multidimensional constructs, only a relative majority of the reviewed studies were based on a multidimensional and complex construct, and virtually no studies integrated findings based on multidimensional methods of operationalization of shame. Notably, most studies referred to the importance of shame regulation but could not or did not provide systematic reviews or meta‐analytic estimates of links between shame regulation and relevant constructs. In this context, the general lack of assessment instruments for adaptive or functional forms of shame appeared to represent an important weakness in the shame literature. The lion's share of findings concerns state and trait shame or shame‐proneness. Such inconsistencies between theoretical and empirical work, as well as within empirical works, make it challenging to synthesize existing knowledge while remaining true to the complexity of shame experience and regulation as conceptually and clinically evident.

In a tentative search of consensus across studies on the definition of shame, some dimensions appeared to be most commonly reported, namely, characterological, behavioural or physical shame, as well as internal and external shame. Regarding shame regulation, Nathanson's (1992) compass of shame model encompassing the shame‐coping strategies of avoidance, withdrawal, attack on self and attack on others was the most common. Among available assessment tools, a wide variety of measures could be identified, with the TOSCA being the most widely used, and notably, many measures to assess shame in relation to specific problem areas were retrieved. By and large, the present umbrella review could at best identify a partial and fairly minor coherence between the conceptualization of shame and its measurement across studies, likely indicating difficulties in reaching consensus on definitions and in operationalizing this construct.

Although a secondary aim of the present umbrella review, a synthesis of the included studies made it possible to corroborate the association between shame and several forms of psychopathology, especially eating disorders, anxiety disorder, PTSD and dissociation symptoms. Furthermore, it was possible to appreciate how shame represents a promising treatment target in clinical practice with different forms of psychopathology. Of note, synthesis studies on the role of shame and shame regulation in externalizing forms of psychopathology remain scarce (Elison et al. 2014; Velotti et al. 2014).

In summary, the results of the present umbrella review provide some support for the multidimensional nature of shame, but further research is needed to understand its complexity and to promote the use of tools that are sensitive to the various aspects it encompasses, including shame regulation beyond the experience of shame. Greater attention to the construct and its operationalization seems required as it would be useful for theoretical advancement and clinical application. In fact, considering its clinical importance, an agreement on the conceptualization of the construct of shame is deemed necessary and would certainly be helpful for practitioners in their daily clinical practice. Given the observed mismatch in conceptualizations, as well as between conceptualizations and measurements, of shame, it is desirable that future research will focus on shame tools that can capture its multidimensionality and facilitate translation to clinical practice.

## Conflicts of Interest

The authors declare no conflicts of interest.

## Data Availability

Data sharing is not applicable to this article as no datasets were generated or analysed during the current study.
